# What Do We Know About Non-Native, Invasive, and Transplanted Aquatic Mollusks in South America?

**DOI:** 10.3390/biology14020151

**Published:** 2025-02-01

**Authors:** Gustavo Darrigran, Carlos Belz, Alvar Carranza, Gonzalo A. Collado, Modesto Correoso, Alejandra A. Fabres, Diego E. Gutiérrez Gregoric, César Lodeiros, Guido Pastorino, Pablo E. Penchaszadeh, Rodrigo B. Salvador, Sonia Santos, Silvana Thiengo, Cristina Damborenea

**Affiliations:** 1 Division Zoología Invertebrados, Museo de La Plata, FCNyM—CONICET, Paseo del Bosque s/n, La Plata 1900, Argentina; invasion@fcnym.unlp.edu.ar (G.D.); dieguty@fcnym.unlp.edu.ar (D.E.G.G.); 2 Laboratorio de Ecologia Aplicada e Bioinvasoes, Centro, Estudos do Mar/Universidade Federal do Parana, Pontal do Parana 83255-976, Brazil; belzoceanos@gmail.com; 3 Departamento de Ecología y Gestión Ambiental, Centro Universitario Regional Este (CURE), Sede Maldonado, Universidad de la República, Maldonado 20100, Uruguay; alvar.carranza@gmail.com; 4 Departamento de Ciencias Básicas, Facultad de Ciencias, Universidad del Bío-Bío, Av. Andrés Bello 720, Chillán 3780000, Chile; gcollado@ubiobio.cl; 5 Departamento Ciencias de la Tierra y la Construcción, Universidad de la Fuerzas Armadas ESPE, Av. General Rumiñahui s/n, Sangolquí 171103, Ecuador; mangelcorreoso@yahoo.com; 6 Department of Biological Sciences, Auburn University, Auburn, AL 36849, USA; alejandra.fabres@gmail.com; 7 Departamento de Acuicultura, Pesca y Recursos Renovables, Facultad de Acuicultura y Ciencias del Mar, Universidad Técnica de Manabí, Bahía de Caráquez EC131450, Ecuador; cesarlodeirosseijo@yahoo.es; 8 Instituto Oceanográfico de Venezuela, Universidad de Oriente, Cumaná 6101, Sucre, Venezuela; 9 Laboratorio de Ecosistemas Costeros-Malacología, Museo Argentino de Ciencias Naturales CONICET, Ciudad Autónoma de Buenos Aires 1405, Argentina; gpastorino@macn.gov.ar (G.P.); pablopench@gmail.com (P.E.P.); 10 Finnish Museum of Natural History, University of Helsinki, Pohjoinen Rautatiekatu 13, 00100 Helsinki, Finland; salvador.rodrigo.b@gmail.com; 11 Departamento de Zoologia, Universidade do Estado do Rio de Janeiro (UERJ), Rio de Janeiro 20550-900, Brazil; gundlachia@yahoo.com.br; 12 Laboratório de Referencia Nacional para Esquistossomose, Malacologia, Instituto Oswaldo Cruz, Fiocruz, Rio de Janeiro 21040-360, Brazil; scarvalhothiengo@gmail.com

**Keywords:** non-native mollusk, introduced mollusk, distribution, impact, spread, effect

## Abstract

Global awareness of introduced species as one of the primary drivers of biodiversity change—causing environmental impacts, and economic and social effects—emphasizes the need to enhance our understanding of these species. Developing a comprehensive database will enable policymakers to identify global bioinvasion patterns and strengthen their capacity to manage them effectively. Aquatic mollusks play a crucial role in the ecosystems they inhabit, influencing food webs and nutrient cycling, and habitat formation and modification. They are also the dominant group in aquaculture, contributing significantly to the economy while also causing economic losses through macrofouling and posing health risks. Despite their importance, information on the introduction, establishment, and dispersal of mollusk species in South America remains scarce and is often confined to the grey literature. With the aim of organizing, increasing, and strengthening the knowledge of non-native and transplanted mollusks in general and aquatic mollusks in particular, 29 specialists in the introduced mollusks of South America, from seven countries, have been working collaboratively since 2016. Each member contributes expertise, data, and bibliographic resources to build the status of the introduced mollusks in South America and provide critical information to prevent future introductions and transplants. In aquatic environments, 41 non-native mollusk species and 18 transplanted species have been identified. Among them, the bivalve *Limnoperna fortunei* stands out with the greatest economic effect, while the gastropods Lymnaeidae and Thiaridae represent significant health concerns. Although this research represents a major step forward, it also highlights challenges such as the scarcity of taxonomic studies and the limited investigation of vast areas in South America. The information compiled in this review serves as a resource for researchers, policymakers, and the general public when addressing mollusk bioinvasions in South America.

## Simple Summary

Introduced species are a major driver of biodiversity loss, with significant impacts on the environment and effects on the economy and human well-being. Aquatic mollusks play a vital role in ecosystems by influencing food webs and nutrient cycling. They also hold economic importance—positively, through aquaculture, and negatively, as contributors to macrofouling. Since 2016, a team of 29 experts from seven South American countries has collaborated to study and manage introduced species. Their research has identified 41 non-native and 18 transplanted aquatic mollusk species, contributing to a deeper understanding of mollusk bioinvasions in South America.

## 1. Introduction

The pace of global change in nature over the past 50 years is unprecedented in human history [1]. The recent Global Assessment Report on Biodiversity and Ecosystem Services by the Intergovernmental Science–Policy Platform on Biodiversity and Ecosystem Services (IPBESs) identifies five direct drivers of this change: changes in land and water, the direct exploitation of organisms, climate change, pollution, and the invasion of non-native species [2]. These are driven by several indirect factors (e.g., social values and behaviors in the patterns of production and consumption, human population dynamics and trends, and the trade market). The rate of change for both the direct and indirect drivers varies across regions and countries [1].

Biological invasions are among the greatest threats to biodiversity, ecosystem functions, and human well-being [3]. The introduction of non-native species into aquatic ecosystems can disrupt ecological processes while also incurring significant economic and social costs [4].

In contrast to the commonly held perspectives on this topic, some researchers, such as Schlaepfer [5], argue that the contributions of non-native species to biodiversity and ecosystem services are often underestimated. According to Schlaepfer [5], biodiversity and sustainability indices should include all of the species in an ecosystem (both native and non-native), emphasizing the intrinsic link between sustainable human well-being and the benefits provided by nature as a whole.

Regardless of their impacts on biodiversity, the introduction of non-native species, has increased in recent decades due to two major global factors [6]: (1) Globalization, a process that began in the mid-1970s and has shaped the current patterns of global trade, increasing the exchanges and interactions between different regions of the planet, thereby facilitating the spread of non-native species [7]; and (2) Global Climate Change, which has altered the environment in ways that often favor non-native species. Many non-native species exhibit greater adaptive and reproductive capacities than native ones, giving them invasive traits, such as the ability to spread and cause environmental impacts, and causing negative socio-economic effects [8].

Mollusks represent the second-largest and most diverse invertebrate phylum, second only to arthropods. They are a vital component of benthic communities, playing a significant role in aquatic ecosystems by functioning and contributing to key ecological processes [9,10]. However, invasive aquatic mollusks can disrupt these processes by altering nutrient dynamics; modifying physical structures and the composition of benthic habitats, consequently affecting native species interactions and food webs; and competing with native species for resources. These impacts can potentially lead to a reduced biomass and to significant changes in community structure [10].

South America is one of the most biodiverse regions in the world. The continent comprises thirteen countries, five of which—Brazil, Colombia, Ecuador, Peru, and Venezuela—are classified as megadiverse [11]. Its vast latitudinal range and diverse climates [12] make it a potential hotspot for non-native species introduction. Nevertheless, South America remains underexplored, and mollusk fauna, both native and non-native, remains understudied [13,14]. The limited knowledge about mollusk population dynamics and distribution, combined with incomplete regional inventories, distorts our understanding of their distribution patterns [15]. This bias arises from two main factors: first, most studies are conducted near research institutions; and second, research priorities often reflect the specific interests of malacologists, focusing on particular environments.

To address these gaps and enhance the understanding of the non-native and transplanted mollusk species in South America, an international academic collaboration group, eMIAS (specialists in introduced mollusks of South America https://emiasgroup.wixsite.com/emias?lang=en, accessed on 30 January 2025), was established in 2016. Comprising 29 malacologists from seven countries (Argentina, Brazil, Chile, Ecuador, Peru, Uruguay, and Venezuela), the group shares information derived from their personal expertise and regional observations, as well as the specialized literature, including the grey literature.

The introduction, establishment, and dispersal of non-native species are closely linked to various socio-economic activities. Non-native species are more likely to establish in areas with significant land-use levels, a high human population density, and robust economic activity [2]. Based on the distribution of non-native mollusk species (NNMS), according to South American ecoregions, together with factors such as urbanization (the presence of cities with more than 500,000 inhabitants), economic activity (airports handling 7 million passengers/year, ports with a TEU value of 700,000, and cargo airports) and conservation status, Darrigran et al. [14] identified four critical zones: the Subtropical Atlantic, the Northern Andes, the Central Andes, and the Southern Andes. These zones are NNMS hotspots, representing the most vulnerable areas to the arrival and establishment of non-native species. Therefore, they should be prioritized for the management of these species.

This study provides an updated assessment of the non-native and invasive mollusks in South America, based on evaluations by regional taxonomic experts. The revised data serve as a vital foundation for developing effective bioinvasion management policies and strategies, while contributing to the identification of global patterns [16]. Additionally, this study offers comprehensive bibliographic resources on non-native and transplanted species, and their effects and impacts in South America.

## 2. Material and Methods

### 2.1. Source

This review incorporates information generated by the eMIAS group, composed of specialists in the introduced mollusks of South America. The data are derived from a compilation and analysis of the recently published literature by the eMIAS members [14,17,18], as well as other recent bibliographic sources. 

For the non-native mollusk species (NNMS) and transplanted native mollusk species (TMS) in South America, nomenclatures, synonyms, and Aphia IDs (unique and stable identifiers for each name in the Aphia platform infrastructure that capture the taxonomic and related data) provided by MolluscaBase [19] were used.

To evaluate the documented natural impacts and socio-economic effects of the NNMS and TMS in South America, the following mutually exclusive categories were used: (1) environmental/biodiversity impacts, (2) economic and social effects, and (3) the effects on human health [18].

### 2.2. Definitions

We consider the following:

*Native species:* defined based on three criteria: (1) they are inherently part of the ecosystem and evolved there; (2) they arrived in the study area long before records were kept; and (3) their arrival occurred naturally, without human assistance [20].
*Non-native or exotic species:* species introduced to new regions through human activities [2].
*Invasive non-native species:* non-native species that have successfully established and spread in a new region, causing impacts on ecosystems and effects on the society [2].
*Biological invasions:* refers to the process involving the intentional or unintentional transport or movement of a species by human activity from its natural range to new regions, where it can be established and spread [2].
*Cryptogenic species*: species for which it is unclear whether their presence in a region is due to natural processes or human intervention [21].
*Transplant (transfer, translocation):* the movement of native species from one locality to another through human action, including a successful establishment outside of their historically known geographical distribution (in this study, within South America). These movements can be accidental or deliberate (e.g., for conservation, trade, or research) [17].
*Species established in the environment:* species with self-sustaining reproductive populations that persist across multiple generations [17].
*Ecoregions:* terrestrial or aquatic areas characterized by a distinct set of natural communities, ecological dynamics, and environmental conditions, typically shared by most of the inhabiting species [22].
*TEU (Twenty-Foot Equivalent Unit):* a standard unit of measurement used in maritime transport to determine a container’s cargo capacity. One TEU corresponds to a container with a load capacity of 28,300 kg [14].

## 3. Results and Discussion

### 3.1. Status and Trends of NNMS in South America

The general knowledge of the non-native species in South America remains limited compared to other regions like Europe and North America [23,24]. Globally, significant information gaps exist across vast areas of South America, particularly in countries such as Bolivia, Guyana, and Belize. These gaps lead to biases in the perceived richness and distribution of NNMS and TMS. Despite these challenges, recent studies at both local and regional scales have focused on the NNMS in South America [14,17,25,26,27,28,29,30,31,32,33,34,35,36].

Currently, 41 aquatic NNMS have been identified in South America, comprising 24 marine species (10 gastropods and 14 bivalves) and 17 freshwater species (12 gastropods and 5 bivalves) (Table 1 and Table 2). Among these, the freshwater clam *Corbicula fluminea* (Müller, 1774), the golden mussel *Limnoperna fortunei* (Dunker, 1857), and the Pacific oyster *Magallana gigas* (Thunberg, 1793) stand out for their significant impacts on native species and ecosystems. These species are classified as aggressive invaders in South America [18,37,38], making them the focus of most regional studies.

The Pacific oyster is particularly notable for its potential role in food security. Initially introduced for aquaculture, it is now cultivated in 33 coastal countries throughout the Americas, including the following seven in South America: Argentina, Brazil, Chile, Colombia, Ecuador, Peru, and Venezuela. While aquaculture operations are absent in Colombia and Venezuela, feral populations have been reported in Argentina and Chile [38]. Beyond its socio-economic benefits, *Magallana gigas* may also fulfill ecological roles previously held by native oysters, whose populations have experienced significant declines.

**Figure 1:**
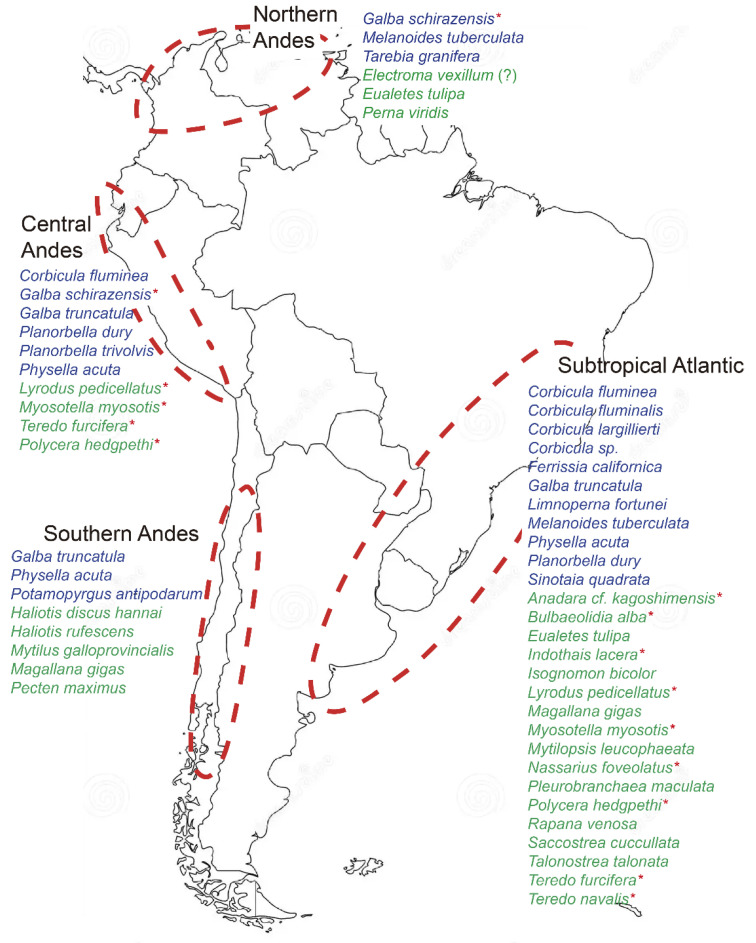
The four zones of South America with the highest number of non-native mollusk species recorded for Darrigran et al. [14]. In blue are the freshwater NNMS and in green are the marine NNMS. The species with red * correspond to the present study.

The findings of this study confirm the results of Darrigran et al. [14], validating the four South American zones with the highest numbers of NNMS: the Subtropical Atlantic, the Northern Andes, the Central Andes, and the Southern Andes (Figure 1), which are related to the presence of large cities, cargo and passenger airports, ports, and the conservation status of the ecoregions of South America. The Subtropical Atlantic area has the highest number of non-native species (28 aquatic species), coinciding with the highest number of large cities, passenger airports, and ports. The Central Andes area has ten aquatic non-native species, the Southern Andes area has eight non-native species, and finally, the Northern Andes area has six non-native species.

At the national level, Ecuador’s continental and insular regions within the Andean ecoregion stand out for their exceptional malacological diversity and high levels of endemism. Although systematic studies comparable to those in Brazil or Argentina are lacking, the region is characterized by rapid colonization processes and an increasing number of recorded invasive species. Identified by Darrigran et al. [14] as part of the Northern Andes zone (Figure 1), this region is highly vulnerable to the introduction and establishment of non-native species, making it a conservation priority. Several studies [14,39,40,41,42,43] suggest the potential for competition with native species. Notably, significant introductions of riverine species through transplantation, such as *Pomacea canaliculata* (Lamarck, 1822), have been documented recently in this region [42,43].

**Table 1:** Aquatic non-native Gastropoda introduced in South America. Aphia ID, unique identifier for each taxon; country codes following ISO 3166 vs. 2020 (https://www.iso.org/iso-3166-country-codes.html, accessed 31 January, 2025); MA, marine species; and FW, freshwater species.

Family	Species	Aphia ID	Geographic Origin	South America Countries	Habitat	References
Aeolidiidae	*Bulbaeolidia alba* (Risbec, 1928)	730446	Indonesia, Japan, Malaysia, Mozambique, Papua Nueva Guinea, Philippines, South Africa, and Tanzania	BR, UY	MA	[44,45]
Ellobiidae	*Myosotella myosotis* (Draparnaud, 1801)	139673	Northeast Atlantic Ocean	PE, UY	MA	[46,47]
Haliotidae	*Haliotis discus hannai* Ino, 1953	397083	East Asia	CH	MA	[14]
	*Haliotis rufescens* Swainson, 1822	445357	Pacific Ocean: USA, Mexico	CH	MA	[14]
Lymnaeidae	*Pseudosuccinea columella* (Say, 1817)	724460	North America, USA	AR, BR, CO, EC, PE, PY, VE, UY	FW	[14]
	*Galba truncatula* (Müller, 1774)	716336	Europe: Germany	AR, BO, BR, CH, CO, EC, PE, VE	FW	[14,48]
	*Galba schirazensis* (Küster, 1862)	716337	Israel, Jordan, Lebanon, Western Syria, southeast Turkey, Iraq, and western Iran	CO, EC, PE, VE	FW	[48,49]
Muricidae	*Indothais lacera* (Born, 1778)	714477	Indo-Pacific, occurring from the Arabian Peninsula to Southeast Asia	BR	MA	[50]
	*Rapana venosa* (Valenciennes, 1846)	140416	Japan Sea, Yellow Sea, Bohai Sea, and East China Sea to Taiwan	AR, BR, UY	MA	[14,51]
Nassariidae	*Nassarius foveolatus* (Dunker, 1847)	560204	Central and East Indian Ocean to East China Sea (Mauritius, Pakistan, India, Myanmar, Thailand, Singapore, Malaysia, and China)	BR	MA	[52]
Physidae	*Physella acuta* (Draparnaud, 1805)	234093	North America	AR, BR, CH, CO, EC, PE, UY	FW	[14,53]
Planorbidae	*Ferrissia californica* (Rowell, 1863)	1001489	North America	BR	FW	[14]
	*Planorbella duryi* (Wetherby, 1879)	1001496	North America: USA.	AR, BR, CO, EC, PE	FW	[14]
	*Planorbella trivolvis* Say, 1817	593137	North America: Canada, USA, Mexico.	BR, EC, PE	FW	[14]
Pleurobran chaeidae	*Pleurobranchaea maculata* (Quoy and Gaimard, 1832)		Australia and New Zealand	AR	MA	[14]
Polyceridae	*Polycera hedgpethi* Er. Marcus, 1964	140835	Indo-Pacific	BR, PE	MA	[54]
Tateidae	*Potamopyrgus antipodarum* (Gray, 1843)	147123	New Zealand	CH	FW	[14]
Thiaridae	*Stenomelania macilenta* (Menke, 1830)	1351381	Asia: Philippines	CH	FW	[14]
	*Melanoides tuberculata* (Müller, 1774)	225694	Africa tropical and subtropical (except for Congo River basin and a big region of the West Coast of Africa) and South Asia	AR, BO, BR, CH, CO, EC, GF, GY, PE, PY, SU, UY, VE	FW	[14,55]
	*Tarebia granifera* (Lamarck, 1816)	397189	Southeast Asia	BR, VE	FW	[14]
Vermetidae	*Eualetes tulipa* (Roseau in Chenu, 1843)	709533	Pacific Ocean: Panama Bay	BR, VE	MA	[14]
Viviparidae	*Sinotaia quadrata* (Benson, 1842)	820921	Asia	AR	FW	[14,31]

**Table 2:** Aquatic non-native Bivalvia introduced in South America. Aphia ID, unique identifier for each taxon; country codes following ISO 3166; MA, marine species; FW, freshwater species; and ?, with doubt.

Family	Species	Aphia ID	Geographic Origin	South America Countries	Habitat	Reference
Arcidae	*Anadara* cf. *kagoshimensis*		Indian Ocean to the western Pacific (India, Sri Lanka, Indonesia, Korea, China, Japan, northern Australia)	UY	MA	[56]
Corbiculidae	*Corbicula fluminea* (Müller, 1774)	181580	Rivers from China and Southeast Asia, Africa, India, Pacific Islands, and Australia	AR, BO, BR, CO, EC, PE, PY, UY, VE	FW	[14]
	*Corbicula fluminalis* (Müller, 1774)	233525	Asia	BR, UY	FW	[14]
	*Corbicula largillierti* (Philippi, 1844)	992070	Asia	AR, BR, PY, UY	FW	[14,33]
	*Corbicula* sp.		Asia	BR	FW	[14]
Dreissenidae	*Mytilopsis leucophaeata* (Conrad, 1831)	156887	Atlantic Ocean: southern coast of eastern United States	BR	MA	[14]
Isognomonidae	*Isognomon bicolor* (C. B. Adams, 1845)	420737	Western Central Atlantic, rocky shores in Bermuda, Florida, Gulf of Mexico, and Caribbean Sea	BR, CO, TT, UY, VE	MA	[14]
Mytilidae	*Arcuatula senhousia* (Benson in Cator, 1842)	505946	Western Pacific Ocean	CO, VE	MA	[14]
	*Limnoperna fortunei* (Dunker, 1857)	506081	China and Southeast Asia	AR, BO, BR, PY, UY	FW	[14]
	*Mytilus galloprovincialis* Lamarck, 1819	140481	Europe: Mediterranean Sea	AR?, BR, CH, UY	MA	[14,57]
	*Perna viridis* (Linnaeus, 1758)	367822	Indo-Pacific Ocean	BR, CO, VE	MA	[14,58]
Teredinidae	*Lyrodus pedicellatus* (Quatrefages, 1849)	141600	Indo-West Pacific Ocean	AR, EC	MA	[59,60,61,62]
	*Teredo furcifera* E. von Martens, 1894	397193	Indo-West Pacific Ocean	AR, BR, EC	MA	[61,62,63]
	*Teredo navalis* Linnaeus, 1758	141607	Northeast Atlantic Ocean?	AR, BR, UY	MA	[62,63,64]
Oetreidae	*Magallana gigas* (Thunberg, 1793)	836033	Japan, Korea, China, and Russia	AR, BR, CH, CO?, EC, PE	MA	[14]
	*Talonostrea talonata* Li & Qui, 1994	506741	China	AR, BR, PE	MA	[14,65]
	*Saccostrea cuccullata* (Born, 1778)	181316	Indo-Pacific	BR	MA	[14,66]
Pectinidae	*Pecten maximus* (Linnaeus, 1758)	140712	Europe and Eastern Atlantic Ocean	CH	MA	[14]
Vulsellidae	*Electroma vexillum* (Reeve, 1857)	234144	Indic Ocean and Red Sea	BR, CO?	MA	[67,68]

**Figure 2:**
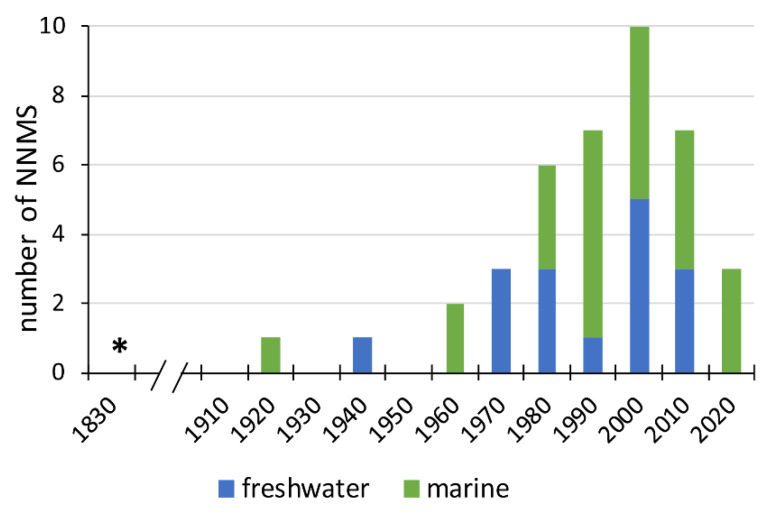
Aquatic NNMS introduced in South America by date (10-year interval), and according to habitat (marine and freshwater). The first recorded NNMS (*) corresponds to a terrestrial species recorded in 1835; the first aquatic NNMS was recorded in the 1920s.

The first records of an introduced mollusk in South America pertain to terrestrial species, with *Bradybaena similaris* (Fërussac, 1821) being the earliest documented case, introduced in Brazil and reported by d’Orbigny in 1835 [14]. The first marine NNMS record corresponds to *Teredo furcifera* reported by E. von Martens (1894) in the 1920s [62]. For freshwater NNMS, *Pseudosuccinea columella* (Say, 1817) was first reported by Morretes in 1949 [69]. Since 1970, the number of introduced aquatic mollusk species in South America has risen sharply (Figure 2). This increase coincides with the expansion of maritime transport, aquaculture, and global trade, which facilitate the introduction of marine and freshwater species worldwide [70,71,72,73].

**Figure 3:**
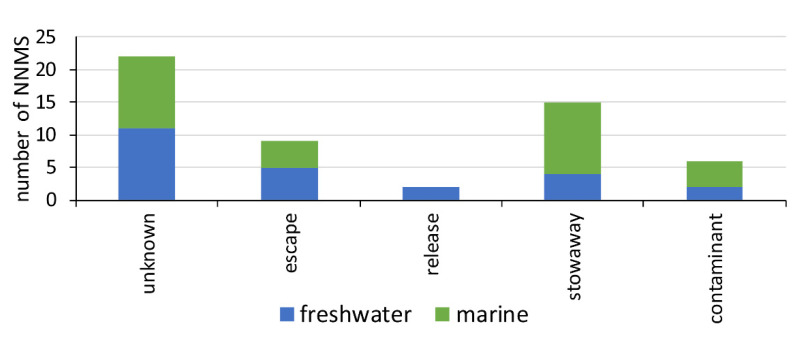
Introduction pathway categories for NNMS in South America. Pathway categories follow Faulkner et al. [74].

In South America, the mollusk introductions have generally been unintentional. Information on the vectors is limited and often unknown (Figure 3). The most common pathway is stowaway transport, which includes ballast water (e.g., *Limnoperna fortunei*), biofouling on ship hulls (e.g., Teredinidae species), and aquaculture activities (e.g., *Magallana gigas*) [14].

### 3.2. Movement of Mollusk Species in South America

Traditionally, the studies on bioinvasion have focused on the species introduced, either deliberately or accidentally, by human activity into biogeographic regions where they were not previously present. Once introduced and established, these species can spread depending on their characteristics and the environment in which they are found. However, a less-studied phenomenon involves the movement of native species within their biogeographic regions due to human intervention, a process known as transplantation, transfer, or translocation [17].

The transplantation of a species beyond its native or historical range of distribution has increased significantly in recent decades [75]. The eMIAS identified 18 aquatic TMS in South America that experienced modifications in their historical distributions within the continent, unrelated to natural dispersal processes. Among these eighteen transplanted species, nine are marine bivalves, two are freshwater bivalves, and seven are freshwater gastropods (Table 3 and Table 4).

To illustrate the changes in mollusk distributions, Darrigran et al. [17] analyzed the percentage of freshwater and marine ecoregions in South America now inhabited by a transplanted species. Their findings revealed that 36% of marine ecoregions and 23% of freshwater ecoregions host transplanted mollusk species. The ecoregions with the greatest number of transplanted species were the Southern Caribbean (three marine species) and the Central Andean Pacific Slopes (three freshwater species). Among freshwater mollusks, the most widely transplanted gastropod is *Pomacea canaliculata*, which inhabits seven ecoregions, while the most frequently transplanted bivalve is the mussel *Leiosolenus aristatus* (Dillwyn, 1817), found in four ecoregions.

**Figure 4:**
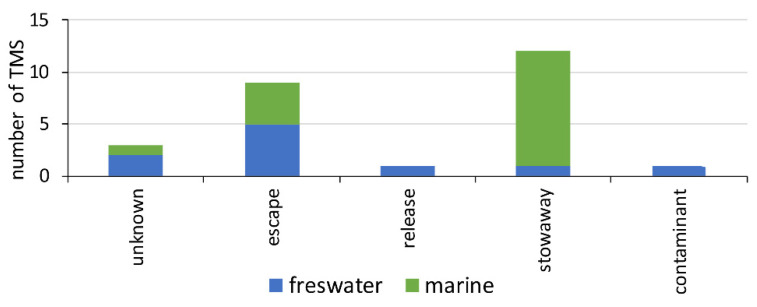
Transplant path categories for NNMS in South America. Categories follow Faulkner et al. [74].

The environmental, economic, and social impacts of transplantation are comparable to those associated with the introduction and establishment of non-native species. The analysis indicates that the most common pathway for transplantation is as stowaways, particularly for bivalves, while escape is a frequent pathway for freshwater species. In South America, TMS transport routes are often linked to economic activities, such as aquaculture and the ornamental species trade [17,76] (Figure 4).

**Table 3:** Transplanted Gastropoda within South America. Aphia ID, unique identifier for each taxon; country codes following ISO 3166; MA, marine species; and FW, freshwater species.

Family	Species	Aphia ID	Known Historical Geographical Distribution	New Locations	Habitat	Reference
Ampullariidae	*Marisa cornuarietis* (Linnaeus, 1758)	737469	CO, VE, TT, and the Amazon Basin regions of BR, BO, and PE.	GY	FW	[77]
	*Pomacea bridgesii* (Reeve, 1856)	741389	Native to the Peruvian and west Amazon.	AR (commercial aquariums). BR (Rio de Janeiro and the Guapimirim), RJ; Paracambi, RJ (22°40.52′ S–43°45.90′ W); Juiz de Fora, MG (21°39′06″ S–43°25′54″ W). CH (commercial aquariums in Santiago de Chile). PE (aquariums and semi-controlled environments). VE (in captivity).	FW	[17]
	*Pomacea canaliculata* (Lamarck, 1822)	741113	South America: Paraná River, Uruguay River, Río de la Plata, and southern Brazil.	AR (Desaguadero, Colorado, South Buenos Aires drainages, and arheic waterbodies). CH (Laguna Conchalí, Los Vilos, IV Region, and Santiago metropolitan region). EC (wide distribution, in 2/3 of the country: Los Ríos, Guayas, Manabí, Esmeraldas, Santo Domingo de los Tsáchilas, Pichincha, Chimborazo, Cotopaxi, Pastaza, Orellana, Morona Santiago, Sucumbíos, and Napo). PE (in coastal locations). VE (in captivity).	FW	[17]
	*Pomacea diffusa* (Blume, 1957)	848365	South America: warm north-central regions, the Amazon basin of BR, BO, and PE, in lentic aquatic environments.	AR (only aquarism). EC (small stream, near the Huapuno river, Arajuno, Pastaza Province, northeast Ecuador). VE (in wildlife and in anthropized environments). UR (aquarism).	FW	[17]
	*Pomacea maculata* (Perry, 1810)	737473	Lower Paraná River, in Plata Basin, AR (southern limit distribution) to north of Manaus in the north of the Amazon basin in BR. It is also present in UR, PY, and PE. It may extend west into BO and EC.	PE (in the north coastal and south-central, transplanted from the Amazon).	FW	[17]
Physidae	*Stenophysa marmorata* (Guilding, 1828)	1253849	Northeastern South America.	EC (Loja city).	FW	[78,79]
Planorbidae	*Drepanotrema surinamense* (Clessin, 1884)	848738	SR, JM.	EC (North Andean Pacific Slopes Ecoregion).	FW	[80]

**Table 4:** Transplanted Bivalvia within South America. Aphia ID, unique identifier for each taxon; country codes following ISO 3166; MA, marine species; and FW, freshwater species.

Family	Species	Aphia ID	Known Historical Geographical Distribution	New Locations	Habitat	References
Dreissenidae	*Mytilopsis* cf. *sallei* (Recluz, 1849)	397147	Central America and Caribbean in coastal lagoons and estuaries. South America in CO and VE.	BR (Recife).	MA	[17]
	*Mytilopsis trautwineana* (Tryon, 1866)	505327	Río San Juan, Choco, CO and Rio Verde, EC. Rivers that drain into the Pacific Ocean.	CO (Rivers draining into the Caribbean coast. Found in shrimp farms of *Litopenaeus vannamei*).	FW	[17]
Micetopodidae	*Anodontites trapesialis* (Lamarck, 1819)	1324136	From MX to UY and AR, except GY, SR, GF, and CH. Mesoamerica, Transandean, Amazonas-Orinoco, Atlantic-Sao Francisco, Parana.	BR (artificial lakes for fish farming, registered in fish farms in Londrina, Paraná, and São Paulo, Santa Catarina, and Amazonas). PE (Piura and Lambayeque).	FW	[17]
Modiolidae	*Modiolus carvalhoi* Klappenbach, 1966	506118	From Rio de Janeiro to Rio Grande, BR.	UY (La Paloma, Rocha).	MA	[81,82,83].
Mytilidae	*Leiosolenus aristatus* (Dillwyn, 1817)	506056	Western Atlantic: North Carolina, USA, to VE including the Gulf of Mexico, BE, Lesser Antilles, CO, PA. Eastern Pacific: from San Diego, California, USA, to Iquique, CH.	BR (Cearà, Baía, Rio de Janeiro, Sao Paulo, Paraná and Santa Catarina, Espiritu Santo). EC (Champion Island, Galapagos).	MA	[17]
	*Mytella strigata* (Hanley, 1843)	1458663	Native in South and Central America, it extends from Bahía Magdalena on the Pacific coast of Baja California Sur (24.6° N) and in the Gulf of California from the north of Guaymas, Sonora, ME (27.9° N) to Cancas, Tumbes, PE (4° S), Galapagos Island, EC. In the Western Atlantic, from the Gulf of Paria, VE, to AR.	CO (expanded—on the basis of genetic studies—from Cartagena Bay). No records of the species in the exhaustive inventories of the Colombian–Venezuelan Caribbean or in studies of benthic communities; mention it in the Bay of Cartagena (Eastern Caribbean), CO, the most northern records in South America. Also, found in artificial systems for shrimp farming in the central-western zone of VE (10°03′05″ N, 65°11′32″ W; West Caribbean)	MA	[17]
	*Mytilus platensis* d’Orbigny, 1842	506158	Western Atlantic from Rio Grande do Sul, BR, to the Golfo San Matías, Chubut, AR.	BR (Bombinhas, Palhoça and Florianópolis, Santa Catarina).	MA	[17]
	*Perna perna* (Linnaeus, 1758)	140483	Most of the coast of BR and VE. It is also found off the coast of Africa.	CH, CO, UY.	MA	[17]
Pectinidae	*Argopecten purpuratus* (Lamarck, 1819)	394269	Pacific coasts of Peru and the North of CH, between Paita (5º S, 81º W) and Valparaíso (33º S, 71º W).	CH (Puerto Montt and Chiloé, X Region, southern Chile).	MA	[17]
Pholadidae	*Barnea truncata* (Say, 1822)	156750	Western Atlantic, from Massachusetts to Texas, USA; CO, VE, and in Bahia Sepetiba, BR.	AR (Bahía Blanca, Buenos Aires).	MA	[17]
Veneridae	*Tawera elliptica* (Lamarck, 1818)	507917	Along both sides of America, from Valparaiso to the Beagle Channel and up to 38° S from the coast of the Atlantic Ocean.	CH (Reñaca in the north of Valparaiso Bay).	MA	[17]

In South America, the only transplanted freshwater bivalve species is *Anodontites trapesialis* (Lamarck, 1819), which is used in aquaculture. Additionally, other gastropods, such as *Pomacea bridgesii* (Reeve, 1856) and *Pomacea lineata* (Spix in Wagner, 1827), are cultivated [84]. Notably, *Pomacea bridgesii* is widely available online as a pet. In Argentina, live specimens of *Pomacea bridgesii* can be purchased through aquarium trade websites that facilitate transactions. The pet trade poses significant challenges as a pathway for invasive non-native species, particularly due to the risks of deliberate or accidental releases. This issue is exacerbated by the profits generated from the pet trade and the strong bonds formed between humans and their pets [85]. Another species, *Pomacea diffusa* Blume, 1957, is widely distributed along the Amazon basin and also appears in the aquarium trade on other continents [86].

It is important to note that the geographical origins of some species remain debated (cryptogenic species), such as *Mytilus platensis* d’Orbigny, 1846, and *Mytilus chilensis* Hupé, 1854 [87,88].

### 3.3. Impacts and Effects of NNMS and TMS in South America

Although species invasions are among the primary drivers impacting biodiversity, community structure and function, ecosystem services, and human health, their impacts on nature and their effects on society remain poorly understood for certain habitats, regions, and taxa [24].

Of the total aquatic NNMS (41 species) and TMS (18 species) recorded, 24 non-native species (9 freshwater species and 15 marine species) and 8 transplanted (3 freshwater and 5 marine) have been documented to cause impacts or effects in South America (Table 5 and Table 6).

Among the marine TMS, all the identified species are bivalves, with five species causing social and economic effects: *Leiosolenus aristatus* (Dillwyn, 1817); *Mytella strigata* (Hanley, 1843); *Mytilopsis trautwineana* (Tryon, 1866); *Argopecten purpuratus* (Lamarck, 1819), and *Tawera elliptica* (Lamarck, 1818). In freshwater systems, the bivalve *Anodontites trapesialis* generates social–economic effects, while the gastropod, *Pomacea canaliculata* impacts in all three categories considered: biodiversity, socio-economic systems, and human health [18].

NNMS impact multiple dimensions. In marine environments, nine species affect biodiversity, nine have social and economic impacts, and two have effects on human health. In freshwater systems, seven NNMS impact biodiversity, seven cause social and economic effects, and four have effects on human health. Among them, the bivalve *Limnoperna fortunei* is the most economically significant species, causing macrofouling in the water intakes of power plants, factories, refrigeration facilities, and water purification plants. The species with the most pronounced effects on human health include *Pseudosuccinea columella* and *Melanoides tuberculata*.

In the marine environment, *Perna viridis* (Linnaeus, 1758) has been a particularly prominent invader since its introduction to South America in Venezuela in 1995 [89]. This species has since spread widely across the continent, reaching the Guanabara Bay in Rio de Janeiro, Brazil, in 2019 [58]. Rapidly expanding through southeastern and southern Brazil, *Perna viridis* has significantly impacted mollusk farming. Along the Brazilian coast, it has been recorded on artificial substrates and aquaculture structures [90,91,92]. With its high invasive potential, *Perna viridis* poses a severe socio-environmental threat, including hybridization with local species and adverse effects on the coastal communities and seafood industries [57].

In summary, 24 NNMS and 8 TMS have documented environmental impacts and socio-economic effects across at least one of the three dimensions considered (Table 5 and Table 6).

**Table 5:** Impacts and effects of freshwater NNMS and TMS in South America, according to the environmental/biodiversity, socio-economic, and public health categories [18].

		Environmental/Biodiversity	Socio-Economic	Public Health
GASTROPODA				
*Galba truncatula* (Lymnaeidae)	NNMS		+	+
*Marisa cornuarietis* (Ampullariidae)	TMS	+	+	+
*Melanoides tuberculata* (Thiaridae)	NNMS	+	+	+
*Physella acuta* (Physidae)	NNMS	+		
*Pomacea canaliculata* (Ampullariidae)	TMS	+	+	+
*Pseudosuccinea columella* (Lymnaeidae)	NNMS		+	+
*Potamopyrgus antipodarum* (Tateidae)	NNMS	+		
*Planorbella trivolvis* (Planorbidae)	NNMS		+	
BIVALVIA				
*Anodontites trapesialis* (Mycetopodidae)	TMS		+	
*Corbicula fluminea* (Cyrenidae)	NNMS	+	+	
*Corbicula largillierti* (Cyrenidae)	NNMS	+	+	
*Limnoperna fortunei* (Mytilidae)	NNMS	+	+	

**Table 6:** Impacts and effects of marine NNMS and TMS in South America, according to the environmental/biodiversity, socio-economic, and public health effects categories. ?, with doubt [18,50,52,93].

		Environmental/Biodiversity	Socio-Economic	Public Health
GASTROPODA				
*Eualetes tulipa* (Vermetidae)	NNMS		+	
*Haliotis discus* (Haliotidae)	NNMS	+	+	
*Haliotis rufescens* (Haliotidae)	NNMS	+	+	+
*Indothais lacera* (Muricidae)	NNMS	+		
*Pleurobranchaea maculata* (Pleurobranchaeidae)	NNMS	+		
*Nassarius foveolatus* (Nassariidae)	NNMS	+?		
*Rapana venosa* (Muricidae)	NNMS	+		
BIVALVIA				
*Argopecten purpuratus* (Pectinidae)	TMS		+	
*Isognomon bicolor* (Isognomonidae)	NNMS	+	+	
*Leiosolenus aristatus* (Mytilidae)	TMS		+	
*Magallana gigas* (Ostreidae)	NNMS	+	+	
*Mytella strigata* (Mytilidae)	TMS		+	
*Mytilopsis leucophaeata* (Dreissenidae)	NNMS		+	
*Mytilopsis trautwineana* (Dreissenidae)	TMS		+	
*Mytilus galloprovincialis* (Mytilidae)	NNMS	+	+	
*Perna viridis* (Mytilidae)	NNMS	+	+	
*Saccostrea cuccullata* (Ostreidae)	NNMS	+		
*Talonostrea talonata* (Ostreidae)	NNMS		+	
*Tawera elliptica* (Veneridae)	TMS	+	+	
*Teredo navalis* (Teredinidae)	NNMS		+	

Large-scale studies conducted by research teams, such as eMIAS, emphasize a lack of societal education and awareness about environmental issues, particularly regarding the impacts of species introductions on biodiversity and the effects on socio-economic systems. This widespread ignorance across many countries has contributed to the presence of inattentive, disengaged, and undertrained officials and policymakers, who are ill equipped to manage non-native species and biodiversity effectively. Consequently, international efforts to address these challenges are either absent or insufficiently coordinated.

In light of this, various authors argue that protecting biodiversity must become a global priority. Safeguarding ecosystems from the increasing impacts of biological invasions is critical, requiring the expansion of protected areas and the allocation of funding commensurate with their significance for humanity’s future on the planet [93,94].

## 4. Conclusions

In recent years, the eMIAS specialist group has established critical foundational knowledge on the non-native and transplanted mollusk species in South America. This information is essential for developing effective policies to manage these species and other biological invasions, as well as for understanding their environmental impacts and socio-economic effects.

The movement of species, driven by globalization, poses a significant threat to biodiversity. The profound impacts and effects of translocated species, particularly mollusks, highlight the urgent need for further research on the introduction and translocation of non-native species in South America.

Given the continent’s vast size and heterogeneity, future research should prioritize underexplored regions and focus on assessing the impacts of non-native and translocated mollusks. This includes analyzing their economic implications, effects on human and veterinary health, and environmental consequences.

Although some isolated efforts have been made to protect South America’s ecosystems from the growing impacts of biological invasions, these initiatives remain insufficient. Research groups across the subcontinent have generated valuable knowledge and foundational data for managing transplanted, non-native, and invasive species. However, despite the presence of centers of expertise and occasional state managers who apply this knowledge effectively, the lack of widespread education of society has hindered the implementation of clear, evidence-based, and sustained environmental policies. Without significant policy reforms and broader societal engagement, South American ecosystems face an increasingly likely future dominated by non-native species, leading to homogenized and degraded environments.

## Data Availability

The raw data supporting the conclusions of this article will be made available by the authors upon request.
